# Supplementary material for: Micro-Structured Two-Component 3D Metamaterials with Negative Thermal-Expansion Coefficient from Positive Constituents
Source: Sci Rep. 2017 Jan 12;7:40643. doi: 10.1038/srep40643 (PMC5228033; doi:10.1038/srep40643)
Supplement: Supplementary Information [file srep40643-s1.pdf]

Supplementary information

Micro-Structured Two-Component 3D Metamaterials with Negative Thermal-Expansion Coefficient from Positive Constituents

Jingyuan Qu[1,2], Muamer Kadic[1,2], Andreas Naber[1], and Martin Wegener[1,2]

[1] *Institute of Applied Physics, Karlsruhe Institute of Technology (KIT), 76128 Karlsruhe, Germany*

[2] *Institute of Nanotechnology, Karlsruhe Institute of Technology (KIT), 76128 Karlsruhe, Germany*

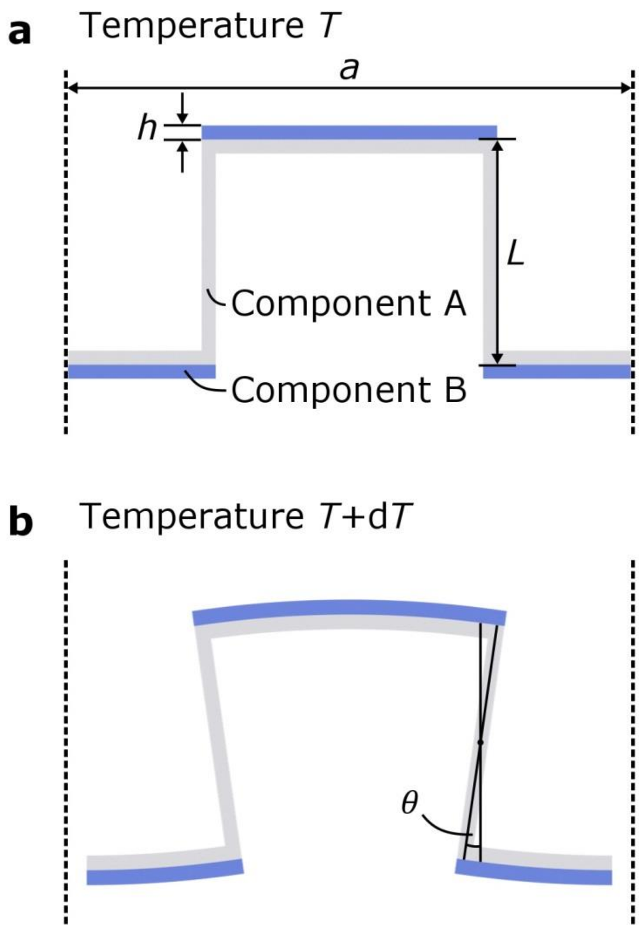

Figure S1 | Design principle of tailored thermal expansion. (a) Basic element of the structure shown in Fig. 1a (also see plane P_1 in Fig. 1c) at a reference temperature T (room temperature). **(b)** Same, but at elevated temperature $T + \mathrm{d}T$ (exaggerated for clarity). The effective thermal length-expansion coefficient of this element, which changes its lattice constant a along one direction, is given by $\alpha_L = \frac{1}{a}\frac{\mathrm{d}a}{\mathrm{d}T}$. Following Ref. [1], translating to our nomenclature, and considering the special case of $h = h_\mathrm{A} = h_\mathrm{B}$ (see Fig. 1), we obtain

$$\alpha_L = \langle \alpha_L \rangle + \frac{2L}{a}\frac{\mathrm{d}}{\mathrm{d}T}\sin\theta(T)$$

$$= \langle \alpha_L \rangle + \left(\alpha_L^\mathrm{A} - \alpha_L^\mathrm{B}\right)\frac{6L}{h}\frac{1}{12 + \left(1 + \frac{E_\mathrm{A}}{E_\mathrm{B}}\right)\left(1 + \frac{E_\mathrm{B}}{E_\mathrm{A}}\right)}.$$

Here, $\langle \alpha_L \rangle$ is the average thermal length-expansion coefficient resulting from the expansion of the bi-material beams alone. For positive constituents, this average is also positive. The second contribution results from the structure and the bending of the beams. For $\left(\alpha_L^\mathrm{A} - \alpha_L^\mathrm{B}\right) < 0$, it is a shrinkage contribution. As pointed out by Lakes [1], the influence of the ratio of Young's moduli $E_\mathrm{A}/E_\mathrm{B}$ is not large. This finding agrees well with the more complete three-dimensional numerical calculations depicted in Supplementary Fig. S3. There, we also find a linear dependence of α_L versus α_L^B. For our parameters, the simple formula for the one-dimensional case overestimates the shrinkage contribution by about 20% with respect to the numerical calculations for the isotropic three-dimensional case.

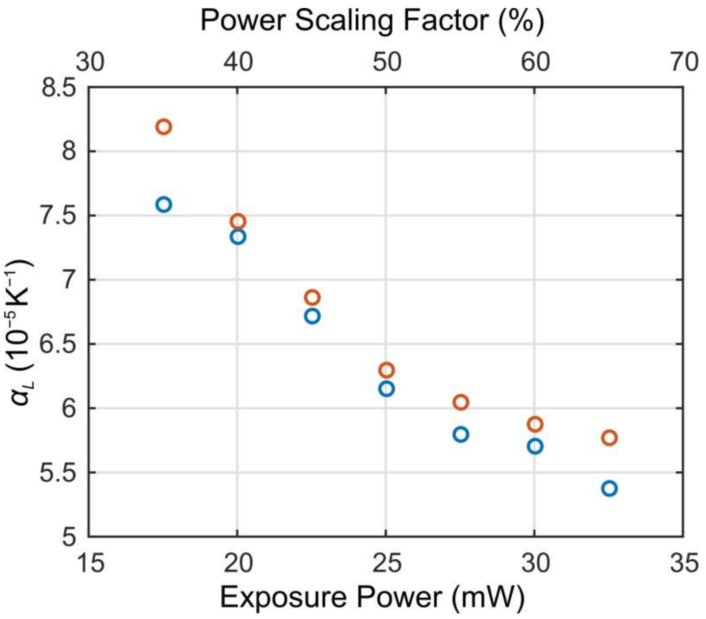

Figure S2 | Bulk thermal expansion coefficients. Measured thermal length-expansion coefficient of bulk polymer cube samples (made from IP-Dip, Nanoscribe GmbH) versus laser exposure power (lower horizontal scale). The corresponding power scaling factor is given on the upper horizontal scale. Results from two different sets of samples and measurements are shown (red and blue). The bulk polymer thermal length-expansion coefficient decreases with increasing exposure power due to increased polymer cross-linking density. For comparison, the single-component microstructure shown in Fig. 3a has been written with a power scaling factor of 65% (also see Methods).

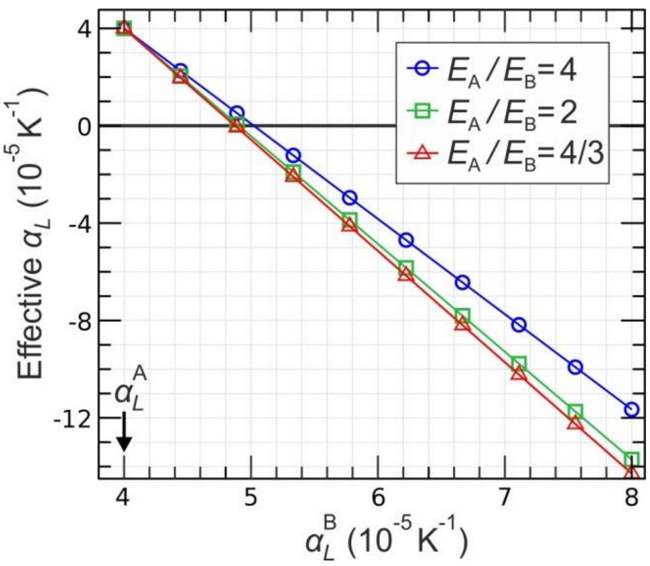

Figure S3 | Tailorable thermal expansion. Calculated effective metamaterial thermal length-expansion coefficient α_L versus the thermal length-expansion coefficient of constituent material **B**, α_L^{B}. The thermal length-expansion coefficient of constituent material **A** is indicated by the arrow. It has been fixed to $\alpha_L^{\mathrm{A}} = +4 \times 10^{-5}\ \mathrm{K}^{-1}$. The ratio of the Young's moduli E of the two constituent materials is the parameter (see legend). The Young's modulus of constituent material **A** has been fixed to $E_{\mathrm{A}} = 4\ \mathrm{GPa}$. Note the sign reversal of α_L around $\alpha_L^{\mathrm{B}} \approx +5 \times 10^{-5}\ \mathrm{K}^{-1}$. Geometrical parameters (compare Fig. 1a): $a = 100\ \mu\mathrm{m}$, $h = 2.5\ \mu\mathrm{m}$, and $L = 40\ \mu\mathrm{m}$.

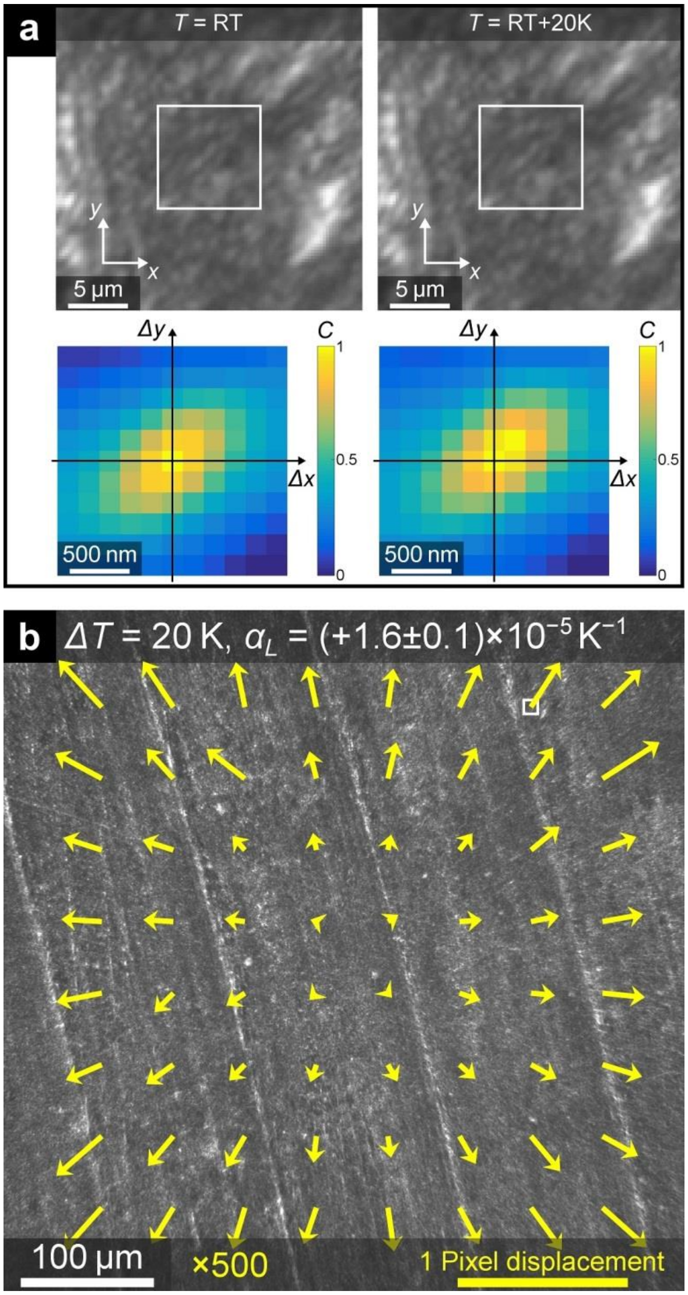

Figure S4 | Illustration of the image cross-correlation analysis. (a) Two optical images of a sample are taken, one reference at room temperature RT (left top), the other one at $\mathrm{RT} + \Delta T$ (right top). Here, the sample is a piece of copper. The two images may appear equal at first sight, but they are actually different. Regions of interest (ROI) at center coordinates (x, y) with a footprint of 33×33 image pixels are defined. One example for a ROI is indicated by the white square. Next, cross-correlation functions

$C(\Delta x, \Delta y)$ of the reference image and ROI are computed. If one correlates the reference with the ROI of the reference (bottom left), one essentially gets the autocorrelation function, which peaks at $(\Delta x = 0, \Delta y = 0)$ indicated by the crossing of the black lines. If one correlates the reference with the ROI of the image at elevated temperature (bottom right), the peak shifts to the displaced position $\left(\Delta x = u_x, \Delta y = u_y\right) = \vec{u} \neq \vec{0}$. Repeating this position for many different ROI, we obtain the displacement vector field $\vec{u}(x, y)$. To remove overall shifts and drifts, we replace $\vec{u}(x, y) \rightarrow \vec{u}(x, y) - \langle\vec{u}\rangle$, where $\langle \dots \rangle$ refers to the average over all chosen ROI. **(b)** Resulting displacement-vector field. The example ROI used in Fig. S4a is shown by the white square in the upper right-hand side corner. To make the yellow displacement vectors $\vec{u}$ visible, we have stretched them by a factor of 500, as indicated at the bottom. To emphasize the sensitivity of the approach, the size of one image pixel (stretched by the same factor of 500) is highlighted by the yellow bar. The derived average length-expansion coefficient of $\alpha_L = (+1.6 \pm 0.1) \times 10^{-5}\ \mathrm{K}^{-1}$ is in agreement with literature.
